# Tracing Antibiotic Resistance Genes along the Irrigation Water Chain to Chive: Does Tap or Surface Water Make a Difference?

**DOI:** 10.3390/antibiotics10091100

**Published:** 2021-09-11

**Authors:** Maria-Theresia Gekenidis, Fiona Walsh, David Drissner

**Affiliations:** 1 Research Division Food Microbial Systems, Agroscope, 8820 Waedenswil, Switzerland; 2 Department of Biology, Maynooth University, W23 F2H6 Maynooth, Ireland; Fiona.Walsh@mu.ie; 3 Department of Life Sciences, Albstadt-Sigmaringen University, 72488 Sigmaringen, Germany; drissner@hs-albsig.de

**Keywords:** antibiotic resistance genes, qPCR, irrigation water chain, surface water, chive, fresh produce

## Abstract

Irrigation water is well known as potential source of pathogens in fresh produce. However, its role in transferring antibiotic resistance determinants is less well investigated. Therefore, we analyzed the contribution of surface and tap water to the resistome of overhead-irrigated chive plants. Field-grown chive was irrigated with either surface water (R-system) or tap water (D-system), from planting to harvest. Water along the two irrigation chains as well as the respective plants were repeatedly sampled and screened for 264 antibiotic resistance genes (ARGs) and mobile genetic elements (MGEs), using high-capacity qPCR. Differentially abundant (DA) ARGs were determined by comparing the two systems. On R-chive, β-lactam ARGs, multidrug-resistance (MDR) determinants, and MGEs were most abundant, while D-chive featured DA ARGs from the vancomycin class. Diversity and number of DA ARGs was the highest on young chives, strongly diminished at harvest, and increased again at the end of shelf life. Most ARGs highly enriched on R- compared to D-chive were also enriched in R- compared to D-sprinkler water, indicating that water played a major role in ARG enrichment. Of note, *bla*_KPC_ was detected at high levels in surface water and chive. We conclude that water quality significantly affects the resistome of the irrigated produce.

## 1. Introduction

After their discovery in the 1920s, antibiotics started revolutionizing medicine and have since saved millions of lives. However, as Alexander Fleming predicted in his Nobel Prize speech, antibiotic resistance in pathogens arose quickly. On average, antibiotic resistance has been identified within 5 to 10 years after introduction of a new antibiotic, but in some cases, even before the antibiotic’s broad clinical application [1]. The development and acquisition of antibiotic resistance in bacteria is a natural defense mechanism. This is why antibiotic-resistant bacteria (ARB) and genes conferring antibiotic resistance (ARGs) have been detected even in environments completely lacking anthropogenic influence, such as permafrost sediments [2]. Nevertheless, the broad application of antibiotics in clinics as well as animal husbandry has promoted the transfer of environmental ARGs into pathogenic bacteria, resulting in many new multidrug-resistant (MDR) pathogens, for some of which only few, if any, last resort antibiotics have remained as treatment options [3].

The dissemination of above-mentioned MDR bacteria and their resistance determinants into the environment has been observed increasingly in the past two decades. For instance, extended-spectrum β-lactamase (ESBL)-producing *Escherichia coli* (*E. coli*) have been detected in wildlife no earlier than 2006 [4], whereas first mentions of bacteria being able to create transferable resistance to extended-spectrum β-lactam antibiotics date back to 1987 [5], and detection of ESBL-producing *Enterobacteriaceae* had already become a clinical routine in 1992 [6]. This highlights the importance of monitoring the dissemination and movement of ARB as well as ARGs in the environment, especially focusing on environments with which humans and their companion animals or livestock come into direct contact [7]. Such environments include playgrounds, pastures, and recreational waters, such as rivers or lakes, which in turn can come into contact with food production systems. Foods and beverages therefore need to be included in monitoring programs as well.

The presence of ARB and ARGs in various food categories has been well documented: in meat and its derivatives; in raw milk and milk products; and in fresh produce, such as ready-to-eat salads and fresh fruits [8,9,10,11,12]. Fresh produce is of special concern, since it is consumed raw or minimally processed, which aids the transfer of ARB and ARGs from the food to the consumer. Moreover, it is often exposed to a variety of potential contamination sources during its growth. These sources range from soil, fertilizer, and irrigation water, workers and their equipment, to various animals [13]. Once introduced into a cultivation system, ARGs can persist for many weeks on the plants and in soil [14,15]. Tracing the origin of ARGs detected on fresh produce to their source can help decrease their abundance on edible plant parts by minimizing their contact with the main sources of contamination.

An important distinction of antibiotic resistance includes whether it is intrinsic or acquired [16]. In the first case, resistance is a bacterial trait and there is no need for mutating or gaining further genes. Therefore, intrinsic resistance cannot be spread in bacterial communities. Acquired resistance, however, can spread horizontally between bacteria and thus from clinically irrelevant, environmental bacteria to pathogens. So-called mobile genetic elements (MGEs), including plasmids, insertion sequences, integrons, or transposons, enable such horizontal gene transfer (HGT) [17]. It is, therefore, equally important to detect and trace MGEs along with clinically relevant ARGs in the environment as well as in foods.

In the present study, we focused our interest on the impact of irrigation water quality on the resistome of fresh produce, as irrigation water quality varies a lot worldwide, from drinking water over ground or surface waters to reclaimed wastewater [18,19,20,21]. As a model plant, we chose greenhouse-grown chive that is irrigated overhead as standard agricultural practice throughout its growth period and thereby has maximum water contact. Two types of water were used in two separate irrigation systems, each irrigating one half of the plants. (1) Tap water was stored in a tank and sterile filtered before drizzling from a sprinkler system onto the plants, and (2) rain and drainage water from agricultural fields and greenhouse rooftops (in the following for simplicity called “surface water”), was draining into a large open-top reservoir, from which it was pumped through a carbon-based particle filter to the inlet of the greenhouse and then into the sprinkler system and onto the plants. We hypothesized that, despite the presence of a multitude of other contamination sources, irrigation water quality could make a difference to the chive’s resistome. To detect and quantify ARGs and MGEs in DNA extracts from water and chive plants, a high-capacity real-time PCR (qPCR) targeting all major antibiotic classes was used, including a total of 264 unique ARGs and MGEs [22].

## 2. Results

### 2.1. Comparing Reservoir- to Tap Water-Irrigated Chive

To compare ARG abundance between chive plants irrigated with either open-top reservoir water (R-chive) or sterile filtered tap water (D-chive), fold changes were calculated using D-chive as a reference. Ideally, a fold change of one indicates equal abundance on the two chive types. However, the equal abundance threshold slightly deviated from one due to differing abundance of 16S rRNA gene copies in the two chive types. Therefore, the equal abundance threshold was indicated in the corresponding figures.

**Figure 1:**
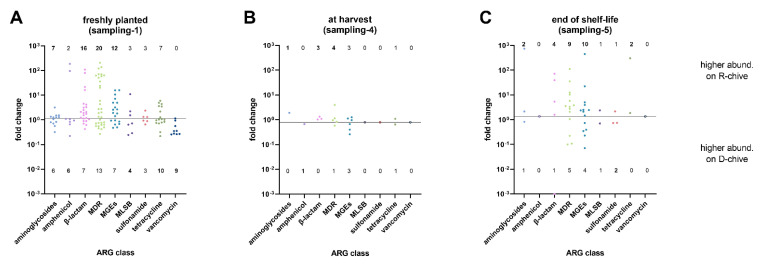
Fold changes of ARGs grouped by class, comparing reservoir water chive (R-chive) to tap water chive (D-chive) at three sampling time points: (**A**) freshly planted chive, (**B**) chive at harvest, and (**C**) chive at the end of its shelf life. The horizontal line marks equal abundance on both chive types. Dots above the line indicate ARGs with higher abundance on R-chive, while dots below the line indicate ARGs with higher abundance on D-chive. Numbers above and below the dots indicate counts of ARGs per class with higher abundance on R- or D-chive, respectively. Empty symbols represent ARG classes with no differentially abundant ARG.

Fold changes of detected ARGs—grouped by class—are shown in Figure 1 for the three sampling times. The exact ARGs detected in each class are listed in Supplementary Table S1. Freshly planted chives (sampling-1) overall displayed a high diversity of differentially abundant (DA) ARGs (up to 33 different genes in the MDR class) as compared to chives from the other two samplings. From classes β-lactam, MDR, and MGEs, the majority of DA ARGs was detected on R-chive. Among the most DA ARGs of R-chive were *ampC, bla*_CMY_, *mdtE/F/L*, *acrA/B/F*, and IS6 group transposases (Table S1). For D-chive, class vancomycin was the most distinctive, including nine DA ARGs, while no vancomycin ARG was detected on R-chive (Figure 1). All but one of these ARGs belonged to the *vanC* cluster [23] (Table S1). The most DA ARGs of D-chive, that is, ARGs with the smallest fold changes, further included *tetA/R*, *cmxA*, *matA*/*mel*, and *mefA* (Table S1). Overall, R- and D-chive of sampling-1 differed significantly (*p* = 0.013; Table 1).

At harvest, only a few DA ARGs were detected when comparing the two chive types. In addition, the fold changes were much smaller than in sampling-1. The most abundant ARGs on R-chive and D-chive were *mexF* and IS6 group *tnpA*, respectively (Table S2). Based on the DA ARG pattern, the two chive types did not differ significantly at harvest (Table 1).

At the end of the chive’s shelf life (sampling-5), that is, after 6 days of storage, the two chive types looked more different (Figure 1). Most DA ARGs were detected on R-chive (numbers above horizontal line). Among the most DA ARGs of R-chive were *strB*, *bla*_ACT_, *bla*_KPC_, *tolC*, IS4 group transposases, and *tetE* (Table S3). On the other hand, on D-chive, the main DA ARGs were *bla*_FOX_, *acrA*, IS6 group transposases, and *lmrA* (Table S3). Overall, however, the observed DA ARG pattern did not significantly differentiate the two chive types at the end of their shelf life (Table 1).

### 2.2. Comparing Sprinkler Water to Corresponding Chives

From the whole irrigation chain, sprinkler water was the one getting into direct contact with the harvested chive leaves. We therefore determined DA ARGs in reservoir compared to tap sprinkler water, and compared these to the respective chives from sampling-1 (highest ARG diversity). The DA ARGs for the two water types based on fold changes (control: tap sprinkler water) are listed in Table S4.

To estimate the contribution of water to differential abundance of ARGs on the two chive types, we compared the DA ARGs of D-/R-chive (Table S1) to those of D-/R-sprinkler water (Table S4). From the top 30% DA ARGs of R-chive, that is, the 25 ARGs with highest FC values, 21 (84%) were also detected in R-sprinkler water as DA ARGs. In other words, the majority of ARGs highly enriched on R- compared to D-chive were also enriched in R- compared to D-sprinkler water, indicating that the water played a major role in the increased abundance of these ARGs on R-chive. Overall, more than half (55%) of all R-chive DA ARGs were also among the 65 R-sprinkler water DA ARGs.

As opposed to the R-sprinkler water, D-sprinkler water contained only a few DA ARGs (*n* = 9). Of these nine D-sprinkler water DA ARGs, two were detected among the D-chive DA ARGs. Thus, and little surprisingly, the effect of sterile filtered irrigation water on the chive resistome seems to be minimal.

### 2.3. Relative ARG and MGE Abundance along the Irrigation Chains to Chive

**Figure 2:**
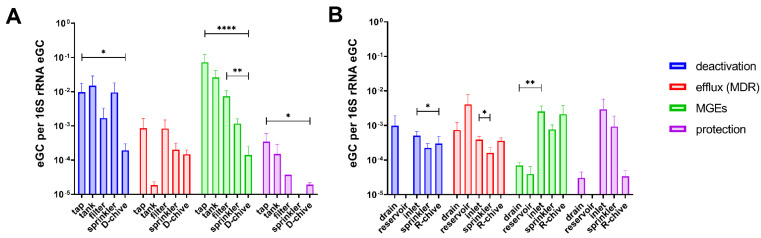
Mean estimated genomic copies (eGC) of ARGs relative to 16S rRNA eGC detected in sampling-1 along the irrigation chains, grouped by ARG function. (**A**) D-system, including tap, tank, sterile filtered, and sprinkler water, as well as D-chive; (**B**) R-system including drain, open-top reservoir, greenhouse inlet, and sprinkler water, as well as R-chive. Statistical significance is indicated: *, *p* < 0.05; **, *p* < 0.01; ****, *p* < 0.0001. Error bars show standard error of the mean.

In order to follow ARGs/MGEs along the irrigation chain to chive, estimated genomic copies (eGC) were calculated for all samples of sampling-1, relative to 16S rRNA eGC (Figure 2). In the D-system (Figure 2A), relative eGC decreased from tap to chive (significant in three of the four functional classes: deactivation, MGE, and protection). Interestingly, although sterile filtration usually decreased relative eGC in the water, the relative eGC of efflux (MDR) class ARGs had increased after filtration of the tank water (Figure 2A).

In the R-system, relative eGC generally decreased from drain to reservoir, with the exception of efflux (MDR) class ARGs that were detected at higher relative eGC in the reservoir compared to the drain water (Figure 2B). As opposed to the D-system, no clear tendency was observed in the R-system from start to end (drain to chive). Relative eGC fluctuated along the irrigation chain and were at similar levels in drain water and chive. Only ARGs belonging to the MGE class were increased on chive as compared to drain water, albeit not significantly.

**Figure 3:**
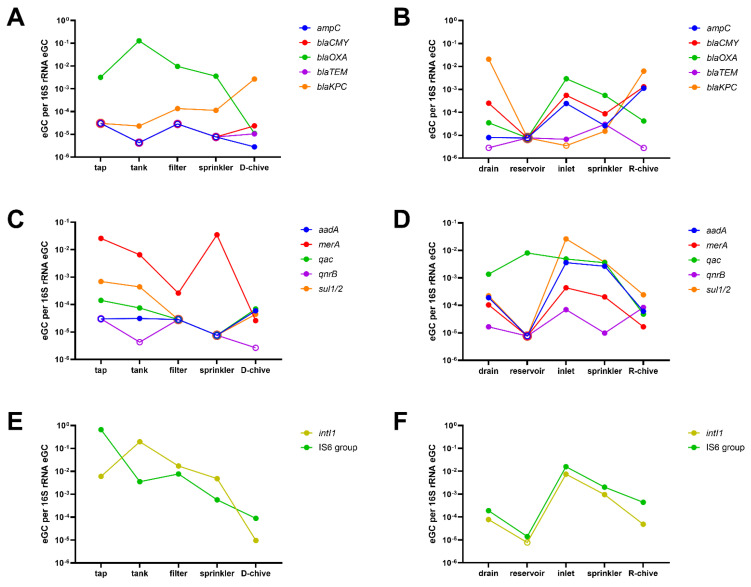
Estimated genomic copies (eGC) of ARGs relative to 16S rRNA eGC detected in sampling-1 along the irrigation chains. Selected single ARGs are displayed. Empty symbols mark ARGs below the limit of detection of the respective sample. (**A**) β-lactam ARGs in the D-system; (**B**) β-lactam ARGs in the R-system; (**C**) other relevant ARGs in the D-system; (**D**) other relevant ARGs in the R-system; (**E**) mobile genetic elements in the D-system; (**F**) mobile genetic elements in the R-system.

Finally, the evolution of selected ARGs and MGEs along the irrigation chain was investigated (Figure 3). In the D-system, as observed overall for the functional classes (Figure 2), relative eGC tended to decrease from tap to chive. An interesting exception is *bla*_KPC_, which increased from tap to chive (Figure 3A). Interestingly, an enrichment of ARGs through the storage of tap water in the plastic tank was rarely observed, namely for *bla*_OXA_ and *intI*1 only (Figure 3A,E, respectively).

In the R-system, reservoir water was poorest in ARGs, although all except *bla*_TEM_ were detected in the inflowing drain water (Figure 3B,D,F), including high relative eGC of the clinically important *bla*_KPC_. Most ARGs were below detection in the reservoir water, except for *qac* and IS6 group transposases, with the latter only marginally above detection (Figure 3D,F, respectively). Relative eGC of most ARGs increased steeply from reservoir to greenhouse inlet water, and was decreased again in sprinkler water (*ampC*, *bla*_CMY_, *bla*_OXA_, *aadA*, *merA*, *sul1*/*2*, *intI*1, and IS6 group transposases). Finally, R-chive generally had lower relative eGC than sprinkler water, with the exception of *ampC*, *bla*_CMY_, *bla*_KPC_, and *qnrB*, which were increased on chive, that is, four of the twelve selected ARGs and MGEs (Figure 3B,D,F).

## 3. Discussion

Irrigation water is one of the main contamination sources in fresh produce cultivation, along with soil and manure [24,25]. Due to their much higher bacterial density, the latter two sources can be expected to contribute significantly more bacteria and ARGs than water to the cultured produce. However, the extreme variations of irrigation water quality worldwide warrant the question whether more highly contaminated water can significantly affect the fresh produce resistome compared to clean water, despite the presence of many other dominant sources of contamination.

We had shown previously that irrigation water quality had a significant impact on the overall detected antibiotic-resistant bacteria in the model system chive irrigated overhead with either sterile filtered tap water (control) or surface water pumped from an open-top reservoir (treatment) [26]. In the present work, we quantified 264 unique MGEs and ARGs—covering all major ARG classes—along the complete irrigation chains and the corresponding chive plants of both the control and the treatment system (D- and R-system, respectively), at three time points. Most DA ARGs were detected on freshly planted chives, and least on chive at harvest. In addition, DA ARG fold changes were generally much higher in young plants than at harvest. The DA ARGs detected when comparing R-chive to D-chive can mostly be attributed to water quality, since irrigation water was the only known factor differentiating the two systems. We assume that the observed irrigation water effect was much more pronounced on young chive plants due to their small size and thereby increased relative contribution of the water, as opposed to the much larger plants at harvest. In young chive, the majority of DA ARGs belonged to the plants irrigated with surface water (R-chive). On the other hand, D-chive clearly stuck out in having nine DA ARGs of the vancomycin class, most belonging to the *vanC* cluster. At harvest, D- and R-chive did hardly differ in terms of DA ARGs. However, the difference between the two chive types became more pronounced during storage with most new DA ARGs belonging to R-chive. This indicates a regrowth of ARG-carrying bacteria during the 6 days, despite the low temperature (4 °C). On R-chive after storage, *bla*_KPC_ was among the most DA ARGs (fold change of 72.189, Table S3). Notably, resistance genes of the *bla*_KPC_ family have become the most important carbapenemases worldwide, which cause great difficulties in managing hospital-acquired infections [27]. The ARG with the highest fold change on D-chive after storage was the AmpC β-lactamase gene *bla*_FOX_, which was not detectable at harvest on the plants but was detected in the water at all stages (tap, tank, filter, and sprinkler). This is another example of an ARG introduced at low levels to the plants, with subsequent strong regrowth of bacterial groups carrying the corresponding gene during storage.

To trace back ARGs with high abundance in one of the two chive types to the respective water, they were compared to DA ARGs in the respective sprinkler water. In control D-sprinkler water, only nine ARGs were differentially abundant compared to R-sprinkler water. Two of these nine were among the D-chive DA ARGs. Thus, for the control system, the differential abundance of 65 ARGs on D-chive compared to R-chive (Figure 1, sampling-1) cannot be explained by their increased abundance in the corresponding sprinkler water. The effect of sterile filtered water on the chive resistome thus seems minimal, and the detection of most D-chive DA ARGs must rely on other, unknown sources. In the R-system, however, of the 70 R-chive DA ARGs, more than half were also differentially abundant in R-sprinkler water. Most interestingly, 21 of the top 25 DA ARGs (that is, the ARGs with the largest fold changes), were also among the DA ARGs of the R-sprinkler water, emphasizing the key role of reservoir water in the increased abundance of the most prominent DA ARGs on the irrigated chive. Irrigation water has been shown previously to be a source of ARB and/or ARGs detected on fresh produce [21,26,28,29]. However, to the best of our knowledge, this is the first study showing a correlation between high differential abundance of ARGs on fresh produce and irrigation water across such a wide array of ARGs.

Measuring estimated genomic copies (eGC) relative to 16S rRNA eGC along the two irrigation chains (sampling-1) revealed that tap water usually had an increased relative eGC compared to D-chive (significant for three of four investigated ARG classes). It seems, therefore, that bacterial communities of tap water contained a higher proportion of ARB than bacterial communities of young chive. For this finding, we could not find a confirmatory or contradicting study either. Sterile filtering the water decreased relative eGC, as expected for most ARG classes, but instead seemed to enrich efflux determinants. Other sterilization methods, such as UV-treatment, have been reported to increase total relative abundance of ARGs in wastewater [30]. On the other hand, ultrafiltration has been shown to have very high ARB and ARG removal efficiencies [31,32], which is, however, more efficient than the filters used in our study.

The closer look at selected ARGs confirmed the findings by ARG class in the D-system. Only *bla*_KPC_ relative eGC slowly increased from tap water to chive (Figure 3A). Finally, storing tap water in a large, non-transparent plastic tank generally did not increase relative eGC of ARGs. This is an interesting observation, since storage of irrigation water in tanks is quite common on fresh produce farms. Unfortunately, we could not find a study on the effect of water storage on ARG concentrations to compare these findings.

The relative eGC per ARG class fluctuated much more along the R-irrigation chain. However, the numbers were comparable between the first and last sampling point (drain water and chive), with the clinically pertinent *bla*_KPC_ reaching top levels in drain water and chive. The only exceptions were MGEs, which were low at the start (drain, reservoir) but increased thereafter (inlet, sprinkler, and chive). From drain to reservoir, we usually observed a clear decrease in relative eGC (exception: efflux-mediating genes). This was quite unexpected, as the water that was draining from the greenhouse rooftops into the open-top reservoir was the only intentionally inflowing water. Additionally, when looking at selected ARGs, the reservoir water was poorest in ARGs, although all except *bla*_TEM_ were clearly detectable in the inflowing drain water. The dilution of ARGs through occasional rainfall is improbable, as such events have been shown to increase rather than decrease ARGs in surface water bodies [33,34]. Wang and colleagues recently made a similar observation of increased ARG abundances in rivers and inlets compared to the receiving lake Honghu [35]. Another study by Di Cesare and coworkers investigated the correlation of various abiotic factors on ARG abundance in lake water. They found that *tetA* and *sul2* genes were positively correlated with dissolved oxygen and negatively to chlorophyll *a* [36]. Since the open-top reservoir investigated in the presented study featured strong growth of a variety of water plants, this might be an important factor explaining the observed strong ARG decrease between drain and reservoir water. However, most ARGs increased again steeply from reservoir to greenhouse inlet, and were only slightly decreased in the downstream sprinklers. Apparently, the long pumping way from the reservoir to the greenhouse allowed regrowth—for example, by attachment and proliferation in biofilms on pipe walls, with subsequent detachment—of resistant bacteria carrying, among other ARGs, *ampC*, *bla*_CMY_, *bla*_OXA_, *aadA*, *merA*, *sul1*/*2*, *intI*1, and IS6 group transposases (Figure 3B,D,F). Of note, agricultural pollution (drainage water) and, to a lesser extent, aerosols (rain water, [37]) can be expected to be the main sources of the ARGs detected in the reservoir water.

Overall, although irrigation water carries low bacterial loads compared to other major contamination sources of fresh produce, we detected a large variety of ARGs with higher abundance on reservoir-irrigated, compared to tap water-irrigated, chive. This irrigation effect was most pronounced on young plants and diminished at harvest, but more pronounced again at the end of the produce’s shelf life. To the best of our knowledge, this is the first study correlating high differential abundance of ARGs on fresh produce to irrigation water. We conclude that irrigation water significantly affects the resistome of irrigated produce. Irrigation water quality should, therefore, not be neglected in favor of other measures taken to reduce ARG transfer from the environment to fresh produce and, ultimately, the consumers.

## 4. Materials and Methods

### 4.1. Experimental Field Trial Setup and Sampling

The setup of the field trial in which irrigation water and chive plant material were collected has been described in detail previously [26]. Briefly, chive plants (*Allium schoenoprasum* L.) were grown on an organic farm in a greenhouse equipped with overhead irrigation pipes. The field was divided into two parts, and plants were irrigated with either surface water pumped from an open-top reservoir according to the standard farm practice (treatment: R-chive) or with sterile filtered tap water (control: D-chive). Chive plants were grown according to Swiss organic farming guidelines (Bio Suisse, [38]). To ensure minimal soil contact, the field beds were covered with an organic foil before planting.

Irrigation water and chive leaves were collected from July to August 2016 in two-week intervals. The complete growth period was covered, that is, eight weeks from seedling planting to harvest of the marketable chives. Special attention was paid to sterile handling, using gloves and sterile sampling utensils and containers for sample collection.

At each sampling, three biological replicates of plant material per condition were collected first. Then, the overhead irrigation systems were started in order to collect water in sterile plastic bottles at different stages in the irrigation chain. For the reservoir-system, water was sampled from (R1) the drain (draining surface water into the open-top reservoir), (R2) the reservoir, (R3) the greenhouse water inlet, and (R4) the corresponding sprinklers. For the tap water system, water was sampled from (D1) the tap, (D2) the reservoir in which the tap water was temporarily stored, (D3) the outlet of a three-stage sterile-filtration unit, and (D4) the corresponding sprinklers. All samples were transported on ice and processed within 10 h (chive) or 24 h (water). Notably, to analyze chive at the end of its shelf life, chive leaves from the last sampling were stored at 4 °C for 6 days and analyzed thereafter.

### 4.2. DNA Extraction and qPCR

To recover DNA from chive plants, 50 g of leaves were gently shaken for 1 min in 300 mL of buffered peptone water (BPW; 10.0 g of peptone, 5.0 g of NaCl, 3.5 g of anhydrous Na_2_HPO_4_, and 1.5 g of KH_2_PO_4_ (Sigma-Aldrich, St. Louis, MI, USA) per 1 L of deionized water, pH 7.0). The suspension was then sonicated for 2 min, and the resulting leaf wash was transferred into sterile 50 mL tubes and centrifuged at 450× *g* for 10 min. The supernatants were discarded and the pellets were stored at −80 °C until further processing.

To recover DNA from water samples, four liters of each sample were pre-filtered using nitrocellulose (NC) filters (5 μm pore size, 45 mm diameter; Merck Millipore, Burlington, MA, USA). Thereof, up to three liters—depending on the cleanness of each pre-filtered water—were filtered using polycarbonate (PC) filters (0.22 µm pore size, 45 mm diameter; Merck Millipore) which were stored at −80 °C until further processing.

DNA extraction was performed using the commercial kits DNeasy PowerPlant and DNeasy PowerWater (Qiagen, Venlo, The Netherlands) for the leaf wash pellets and the PC water filters, respectively. Manufacturer instructions were followed, and DNA quality and quantity were measured using a Quant-iT^TM^ High-Sensitivity dsDNA Assay Kit on a Qubit 3.0 Fluorometer and a NanoDrop^TM^ One Spectrophotometer (Thermo Fisher Scientific, Waltham, MA, USA).

DNA extracts from leaf washes and water samples from sampling-1 (directly after planting), sampling-4 (eight weeks after planting, that is, at harvest), and sampling-5 (chive plants at the end of their shelf life) were shipped on dry ice to Michigan State University for a high-capacity qPCR screening [22]. Using 382 validated primer sets, samples were screened for 264 unique MGEs and ARGs, covering all major ARG classes. Additionally, the 16S rRNA gene was amplified to allow relative ARG quantification and sample comparison. Each sample was measured in triplicate.

### 4.3. Data Analysis

A cycle threshold (C_T_)-value of 30 was used as detection limit (cutoff). Moreover, a gene was considered as present only when at least two of the three technical replicates showed amplification. To compare relative abundance of ARGs or MGEs between samples, the ΔΔC_T_ method was applied according to Livak and Schmittgen [39]:

ΔC_T_ = C_T (ARG/MGE)_ − C_T (16S)_ (1)

ΔΔC_T_ = ΔC_T (treatment)_ − ΔC_T (control)_ (2)

where C_T_ is the cycle threshold (replaced by 30 when below detection), ARG/MGE is each of the 382 amplified genes, 16S is the 16S rRNA gene used for normalization, treatment is each analyzed sample, and control is the sample used as reference. Fold changes (FC) were then calculated as:

FC = 2 ^–^^ΔΔC^_T_ (3)

Finally, assuming perfect qPCR efficiency and a detection limit of 30, estimated genomic copies (eGC) were calculated for each amplified gene using the following equation:

eGC = 10 ^((30-C^_T_^)/3^.^3333)^ (4)

Therefore, relative abundance was calculated by dividing the eGC of each gene by the eGC of the 16S rRNA gene in the same sample. Note that, often, multiple primer pairs existed for one ARG family (e.g., *aadA1*, *aadA2*, *aadA5*, and *aadA9*). In these cases, the sum of the single eGC values was calculated before normalizing by 16S rRNA eGC. When an ARG did not amplify in a sample, a C_T_ of 30 was used to determine the detection limit of that sample in dependence of its 16S rRNA eGC.

### 4.4. Statistics

Significant differences in DA ARGs between R- and D-chive were determined by applying Fisher’s exact test for count data to the contingency tables containing the numbers of DA ARGs detected per sampling for each chive type, split by ARG class.

Significant differences in eGC numbers along the irrigation chains were calculated on log-transformed data using ordinary one-way ANOVA with subsequent Tukey’s multiple comparison test in GraphPad Prism 9.1.2 (GraphPad Software, San Diego, CA, USA). Significant differences were marked in the corresponding figures.

## Figures and Tables

**Table 1:** Contingency tables of DA ARGs detected per sampling on R- and D-chive, split by ARG class. Statistical differences between R- and D-chive per sampling are shown at the bottom, as determined by Fisher’s exact test for count data (alternative hypothesis two-sided). *, *p* < 0.05; ns, not significant.

	Sampling-1		Sampling-4		Sampling-5	
	R-chive	D-chive	R-chive	D-chive	R-chive	D-chive
*aminoglycosides*	7	6	1	0	2	1
*amphenicol*	2	6	0	1	0	0
*β-lactam*	16	7	3	0	4	1
*MDR*	20	13	4	1	9	5
*MGE*	12	7	3	3	10	4
*MLSB*	3	4	0	0	1	1
*sulfonamide*	3	3	0	0	1	2
*tetracycline*	7	10	1	1	2	0
*vancomycin*	0	9	0	0	0	0
*p-value* *(Fisher’s Exact Test)*	0.01302 (*)		0.4605 (*ns*)		0.8739 (*ns*)	

## Data Availability

Data supporting reported results are provided in supplementary Tables S1 to S4 as well as in CHIVE_field-study_qPCR_raw-data.xlsx.

## Supplementary Materials

The following are available online at https://www.mdpi.com/article/10.3390/antibiotics10091100/s1, Table S1: Fold changes of ARGs for R-chive for sampling-1, Table S2: Fold changes of ARGs for R-chive for sampling-4, Table S3: Fold changes of ARGs for R-chive for sampling-5, Table S4: Fold changes of ARGs calculated for R-sprinkler water.

## References

[1] Centers for Disease Control and Prevention (2019). Antibiotic Resistance Threats in the United States.

[2] Mindlin S., Soina V., Petrova M., Gorlenko Z.M. (2008). Isolation of antibiotic resistance bacterial strains from Eastern Siberia permafrost sediments. Russ. J. Genet..

[3] Magiorakos A.P., Srinivasan A., Carey R.B., Carmeli Y., Falagas M.E., Giske C.G., Harbarth S., Hindler J.F., Kahlmeter G., Olsson-Liljequist B. (2012). Multidrug-resistant, extensively drug-resistant and pandrug-resistant bacteria: An international expert proposal for interim standard definitions for acquired resistance. Clin. Microbiol. Infect..

[4] Guenther S., Ewers C., Wieler L.H. (2011). Extended-spectrum beta-lactamases producing *E. coli* in wildlife, yet another form of environmental pollution?. Front. Microbiol..

[5] Neu H. (1987). Penicillin-binding proteins and beta-lactamases: Their effects on the use of cephalosporins and other new beta-lactams. Curr. Clin. Top. Infect. Dis..

[6] Thomson K.S., Hayden M.E., Sanders C.C., Bradford P.A. (1992). Detection of extended spectrum β-lactamases of *Enterobacteriaceae* in routine disk diffusion susceptibility tests. Proceedings of the 92nd Annual Meeting of the American Society for Microbiology.

[7] Thanner S., Drissner D., Walsh F. (2016). Antimicrobial resistance in agriculture. MBio.

[8] Vogt D., Overesch G., Endimiani A., Collaud A., Thomann A., Perreten V. (2014). Occurrence and genetic characteristics of third-generation cephalosporin-resistant *Escherichia coli* in Swiss retail meat. Microb. Drug Resist..

[9] Pesavento G., Calonico C., Ducci B., Magnanini A., Lo Nostro A. (2014). Prevalence and antibiotic resistance of *Enterococcus* spp. isolated from retail cheese, ready-to-eat salads, ham, and raw meat. Food Microbiol..

[10] Geser N., Stephan R., Hächler H. (2012). Occurrence and characteristics of extended-spectrum β-lactamase (ESBL) producing *Enterobacteriaceae* in food producing animals, minced meat and raw milk. BMC Vet. Res..

[11] Blau K., Bettermann A., Jechalke S., Fornefeld E., Vanrobaeys Y., Stalder T., Top E.M., Smalla K. (2018). The Transferable Resistome of Produce. MBio.

[12] Al-Kharousi Z.S., Guizani N., Al-Sadi A.M., Al-Bulushi I.M. (2019). Antibiotic resistance of *Enterobacteriaceae* isolated from fresh fruits and vegetables and characterization of their AmpC β-lactamases. J. Food Prot..

[13] Drissner D., Zuercher U., Motarjemi Y., Todd E., Moy G. (2014). Microbial safety of fresh fruits and vegetables. Encyclopedia of Food Safety.

[14] Gekenidis M.-T., Rigotti S., Hummerjohann J., Walsh F., Drissner D. (2020). Long-Term Persistence of *bla*_CTX-M-15_ in Soil and Lettuce after Introducing Extended-Spectrum β-Lactamase (ESBL)-Producing *Escherichia coli* via Manure or Water. Microorganisms.

[15] Wang F.-H., Qiao M., Su J.-Q., Chen Z., Zhou X., Zhu Y.-G. (2014). High throughput profiling of antibiotic resistance genes in urban park soils with reclaimed water irrigation. Environ. Sci. Technol..

[16] Martinez J.L. (2014). General principles of antibiotic resistance in bacteria. Drug Discov. Today Technol..

[17] De la Cruz F. (2020). Horizontal Gene Transfer: Methods and Protocols.

[18] Ensink J.H.J., Mahmood T., Dalsgaard A. (2007). Wastewater-irrigated vegetables: Market handling versus irrigation water quality. Trop. Med. Int. Health.

[19] Maimon A., Tal A., Friedler E., Gross A. (2010). Safe on-site reuse of greywater for irrigation-a critical review of current guidelines. Environ. Sci. Technol..

[20] Uyttendaele M., Jaykus L.A., Amoah P., Chiodini A., Cunliffe D., Jacxsens L., Holvoet K., Korsten L., Lau M., McClure P. (2015). Microbial hazards in irrigation water: Standards, norms, and testing to manage use of water in fresh produce primary production. Compr. Rev. Food Sci. Food Saf..

[21] Araújo S., Silva I.A.T., Tacão M., Patinha C., Alves A., Henriques I. (2017). Characterization of antibiotic resistant and pathogenic *Escherichia coli* in irrigation water and vegetables in household farms. Int. J. Food Microbiol..

[22] Zhu Y.G., Johnson T.A., Su J.Q., Qiao M., Guo G.X., Stedtfeld R.D., Hashsham S.A., Tiedje J.M. (2013). Diverse and abundant antibiotic resistance genes in Chinese swine farms. Proc. Natl. Acad. Sci. USA.

[23] Panesso D., Abadía-Patiño L., Vanegas N., Reynolds P.E., Courvalin P., Arias C.A. (2005). Transcriptional analysis of the *vanC* cluster from *Enterococcus gallinarum* strains with constitutive and inducible vancomycin resistance. Antimicrob. Agents Chemother..

[24] Alegbeleye O.O., Singleton I., Sant’Ana A.S. (2018). Sources and contamination routes of microbial pathogens to fresh produce during field cultivation: A review. Food Microbiol..

[25] Machado-Moreira B., Richards K., Brennan F., Abram F., Burgess C.M. (2019). Microbial contamination of fresh produce: What, where, and how?. Compr. Rev. Food Sci. Food Saf..

[26] Gekenidis M.T., Schoner U., von Ah U., Schmelcher M., Walsh F., Drissner D. (2018). Tracing back multidrug-resistant bacteria in fresh herb production: From chive to source through the irrigation water chain. FEMS Microbiol. Ecol..

[27] Cuzon G., Naas T., Nordmann P. (2010). KPC carbapenemases: What is at stake in clinical microbiology?. Pathol. Biol..

[28] Holvoet K., Sampers I., Callens B., Dewulf J., Uyttendaele M. (2013). Moderate prevalence of antimicrobial resistance in *Escherichia coli* isolates from lettuce, irrigation water, and soil. Appl. Environ. Microbiol..

[29] Njage P.M., Buys E.M. (2014). Pathogenic and commensal *Escherichia coli* from irrigation water show potential in transmission of extended spectrum and AmpC beta-lactamases determinants to isolates from lettuce. Microb. Biotechnol..

[30] Hu Q., Zhang X.-X., Jia S., Huang K., Tang J., Shi P., Ye L., Ren H. (2016). Metagenomic insights into ultraviolet disinfection effects on antibiotic resistome in biologically treated wastewater. Water Res..

[31] Fatta-Kassinos D., Dionysiou D.D., Kümmerer K. (2015). Advanced Treatment Technologies for Urban Wastewater Reuse.

[32] Wallmann L., Krampe J., Lahnsteiner J., Radu E., van Rensburg P., Slipko K., Wögerbauer M., Kreuzinger N. (2021). Fate and persistence of antibiotic-resistant bacteria and genes through a multi-barrier treatment facility for direct potable reuse. J. Water Reuse Desal..

[33] Di Cesare A., Eckert E.M., Rogora M., Corno G. (2017). Rainfall increases the abundance of antibiotic resistance genes within a riverine microbial community. Environ. Pollut..

[34] Zhang S., Pang S., Wang P., Wang C., Han N., Liu B., Han B., Li Y., Anim-Larbi K. (2016). Antibiotic concentration and antibiotic-resistant bacteria in two shallow urban lakes after stormwater event. Environ. Sci. Pollut. Res..

[35] Wang Z., Han M., Li E., Liu X., Wei H., Yang C., Lu S., Ning K. (2020). Distribution of antibiotic resistance genes in an agriculturally disturbed lake in China: Their links with microbial communities, antibiotics, and water quality. J. Hazard. Mater..

[36] Di Cesare A., Eckert E.M., Teruggi A., Fontaneto D., Bertoni R., Callieri C., Corno G. (2015). Constitutive presence of antibiotic resistance genes within the bacterial community of a large subalpine lake. Mol. Ecol..

[37] Andronache C. (2003). Estimated variability of below-cloud aerosol removal by rainfall for observed aerosol size distributions. Atmos. Chem. Phys..

[38] (2015). Bio Suisse Standards for the Production, Processing and Marketing of ‘bud’ Products.

[39] Schmittgen T.D., Livak K.J. (2008). Analyzing real-time PCR data by the comparative C_T_ method. Nat. Prot..

