# Supplementary material for: Tracing Antibiotic Resistance Genes along the Irrigation Water Chain to Chive: Does Tap or Surface Water Make a Difference?
Source: Antibiotics (Basel). 2021 Sep 11;10(9):1100. doi: 10.3390/antibiotics10091100 (PMC8469318; doi:10.3390/antibiotics10091100)
Supplement: Supplementary file 1 [file antibiotics-10-01100-s001.zip › Supplementary-Tables-Gekenidis-2021.pdf]

Supplementary Tables provided for manuscript *Tracing Antibiotic Resistance Genes along the Irrigation Water Chain to Chive: Does Tap or Surface Water Make a Difference?*

Gekenidis, M.-T.; Walsh, F.; Drissner, D.

Table S1. Fold changes of ARGs calculated for R-chive using D-chive as reference, grouped by antibiotic class. All ARGs displayed in Figure 1 for sampling-1 are listed; a few rare ARGs were therefore omitted from the list. Insertional seq., insertional sequence; plasmid incomp., plasmid incompatibility.

Gene Name	Target Antibiotics (Major)	Functional Classification	Fold Change
aacC2	aminoglycoside	deactivate	0.704
aadA	aminoglycoside	deactivate	0.557
aadA	aminoglycoside	deactivate	3.169
aadA5	aminoglycoside	deactivate	1.342
aadA	aminoglycoside	deactivate	0.319
aadA5	aminoglycoside	deactivate	1.496
aadA	aminoglycoside	deactivate	1.253
aadA	aminoglycoside	deactivate	0.827
aadA9	aminoglycoside	deactivate	1.278
aadA	aminoglycoside	deactivate	1.049
aadA9	aminoglycoside	deactivate	0.797
str	aminoglycoside	deactivate	1.524
strB	aminoglycoside	deactivate	1.232
cmlA	amphenicol	efflux	0.825
floR	amphenicol	efflux	0.942
yidY/mdtL	amphenicol	efflux	0.659
mdtL	amphenicol	efflux	185.878
cmxA	amphenicol	efflux	0.221
catA1	amphenicol	deactivate	1.042
rarD	amphenicol	efflux	1.089
rarD	amphenicol	efflux	95.289
blaACT	β-lactam	deactivate	0.892
ampC	β-lactam	deactivate	16.126
ampC	β-lactam	deactivate	79.731
blaCMY	β-lactam	deactivate	21.359
ampC	β-lactam	deactivate	0.894
blaMOX/blaCMY	β-lactam	deactivate	2.102
blaSHV	β-lactam	deactivate	1.196
bla1	β-lactam	deactivate	1.133
blaOXA	β-lactam	deactivate	3.128
cphA	β-lactam	deactivate	4.479
blaL1	β-lactam	deactivate	3.264
blaCMY	β-lactam	deactivate	108.744
blaACT	β-lactam	deactivate	0.424
blaACT	β-lactam	deactivate	1.921
blaCMY	β-lactam	deactivate	1.725
blaFOX	β-lactam	deactivate	2.079
blaCTX-M	β-lactam	deactivate	1.052
blaCTX-M	β-lactam	deactivate	1.214
blaSFO	β-lactam	deactivate	1.554

mecA	β-lactam	protection	4.126
blaTEM	β-lactam	deactivate	0.615
blaPER	β-lactam	deactivate	0.701
KPC	β-lactam	deactivate	2.500
acrB	MDR	efflux	50.795
acrF	MDR	efflux	120.276
adeA	MDR	efflux	0.499
acrA	MDR	efflux	58.236
acrA	MDR	efflux	20.087
emrD	MDR	efflux	0.421
uidA	MDR	efflux	62.231
mdtE	MDR	efflux	138.788
mdtG	MDR	efflux	69.851
yceL/mdtH	MDR	efflux	0.799
mdtH	MDR	efflux	35.919
mexA	MDR	efflux	1.261
acrR	MDR	regulator	2.020
acrR	MDR	regulator	70.959
qacEΔ1	MDR	efflux	0.559
emrB/qacA	MDR	efflux	0.753
qacEΔ1	MDR	efflux	0.636
oprD	MDR	efflux	0.759
oprJ	MDR	efflux	0.551
ttgA	MDR	efflux	2.788
mepA	MDR	efflux	4.327
mexE	MDR	efflux	1.869
mexF	MDR	efflux	0.451
acrA	MDR	efflux	0.268
acrA	MDR	efflux	1.342
tolC	MDR	efflux	63.455
tolC	MDR	efflux	2.713
tolC	MDR	efflux	5.902
acrR	MDR	regulator	23.844
qacEΔ1	MDR	efflux	0.711
emrB/qacA	MDR	efflux	0.627
marR	MDR	regulator	1.009
mdtF	MDR	efflux	204.883
ISPps	insertional seq.	MGE	2.562
ISSm2	insertional seq.	MGE	0.473
ISAba3	insertional seq.	MGE	0.496
ISEfm1	insertional seq.	MGE	0.948
intI1	integrase	MGE	1.903
intI1	integrase	MGE	2.668
intI3	integrase	MGE	7.168
intI1	integrase	MGE	1.423
intI3	integrase	MGE	4.967
IncN_rep	plasmid incomp.	MGE	0.643
IncP_oriT	plasmid incomp.	MGE	3.205
repA	plasmid replication	MGE	0.931
tetR	plasmid replication	MGE	0.847
IS6 *tnpA*, IS26, ISG 6, Tn24, Tn25	transposase	MGE	10.648
IS6 Group, *tnpA*, IS6100, ISG 6	transposase	MGE	1.809

IS4 Group, *tnpA*, IS4, ISG 4, Tn22	transposase	MGE	0.677
IS21 Group, *tnpA*, Tn21, ISG 21	transposase	MGE	4.928
IS6 Group, IS1216	transposase	MGE	15.879
IS6 Group, *tnpA*	transposase	MGE	15.587
ermF	MLSB	protection	0.636
lmrA	MLSB	efflux	10.904
matA/mel	MLSB	efflux	0.243
mphA	MLSB	deactivate	0.719
mefA	MLSB	efflux	0.287
vgaB	MLSB	efflux	1.531
oleC	MLSB	efflux	2.219
sul2	sulfonamide	protection	1.306
folA	sulfonamide	protection	1.298
folP	sulfonamide	protection	0.931
folP	sulfonamide	protection	0.882
sul3	sulfonamide	protection	0.648
sul1	sulfonamide	protection	2.355
tetX	tetracycline	deactivate	0.778
tetR	tetracycline	regulator	0.317
tetR	tetracycline	regulator	3.624
tetPB	tetracycline	protection	1.063
tetPB	tetracycline	protection	0.827
tetPB	tetracycline	protection	1.029
tetPB	tetracycline	protection	0.712
tetA	tetracycline	efflux	0.218
tetA/B	tetracycline	efflux	4.371
tetC	tetracycline	efflux	4.873
tetG	tetracycline	efflux	0.955
tetG	tetracycline	efflux	1.003
tetA/B	tetracycline	efflux	5.945
tetL	tetracycline	efflux	0.754
tetL	tetracycline	efflux	1.277
tetC	tetracycline	efflux	3.465
tetE	tetracycline	efflux	2.163
vanC	vancomycin	protection	0.260
vanC	vancomycin	protection	0.351
vanTC	vancomycin	protection	0.268
vanRA	vancomycin	protection	1.139
vanC2/vanC3	vancomycin	protection	0.257
vanRC	vancomycin	protection	0.353
vanRC4	vancomycin	protection	0.939
vanSC	vancomycin	protection	0.279
vanTC	vancomycin	protection	0.558

Table S2. Fold changes of ARGs calculated for R-chive using D-chive as reference, grouped by antibiotic class. All ARGs displayed in Figure 1 for sampling-4 are listed; a few rare ARGs were therefore omitted from the list. Insertional seq., insertional sequence; plasmid incomp., plasmid incompatibility.

Gene Name	Target Antibiotics (Major)	Functional Classification	Fold Change
strB	aminoglycoside	deactivate	1.902
cmlA	amphenicol	efflux	0.665
bla1	β-lactam	deactivate	1.084
blaCMY	β-lactam	deactivate	1.327
KPC	β-lactam	deactivate	1.031
emrB/qacA	MDR	efflux	0.829
mexE	MDR	efflux	0.577
mexF	MDR	efflux	3.952
tolC	MDR	efflux	0.951
emrB/qacA	MDR	efflux	1.142
ISPps	insertional seq.	MGE	1.121
IS6 *tnpA*, IS26, ISG 6, Tn24, Tn25	transposase	MGE	0.966
IS6 Group, *tnpA*, IS6100, ISG 6	transposase	MGE	0.666
IS4 Group, *tnpA*, IS4, ISG 4, Tn22	transposase	MGE	1.257
IS6 Group, IS1216	transposase	MGE	0.400
IS6 Group, *tnpA*	transposase	MGE	0.258
tetA/B	tetracycline	efflux	0.644
tetPB	tetracycline	protection	1.082

Table S3. Fold changes of ARGs calculated for R-chive using D-chive as reference, grouped by antibiotic class. All ARGs displayed in Figure 1 for sampling-5 are listed; a few rare ARGs were therefore omitted from the list. Insertional seq., insertional sequence; plasmid incomp., plasmid incompatibility.

**Gene Name**	**Target Antibiotics (Major)**	**Functional Classification**	**Fold Change**
aac(6')-Ib	aminoglycoside	deactivate	0.821
aadA	aminoglycoside	deactivate	2.161
strB	aminoglycoside	deactivate	745.022
blaOXA	β-lactam	deactivate	1.593
blaCMY	β-lactam	deactivate	5.362
blaACT	β-lactam	deactivate	39.447
blaFOX	β-lactam	deactivate	0.001
KPC	β-lactam	deactivate	72.189
emrD	MDR	efflux	1.474
qacEΔ1	MDR	efflux	3.945
qacEΔ1	MDR	efflux	0.071
oprD	MDR	efflux	2.443
oprJ	MDR	efflux	2.379
ttgA	MDR	efflux	2.519
mepA	MDR	efflux	1.063
mexE	MDR	efflux	2.087
mexF	MDR	efflux	3.307
acrA	MDR	efflux	0.223
acrA	MDR	efflux	0.108
tolC	MDR	efflux	35.518
tolC	MDR	efflux	112.163
qacEΔ1	MDR	efflux	3.814
ISPps	insertional seq.	MGE	3.564
ISSm2	insertional seq.	MGE	10.342
ISAba3	insertional seq.	MGE	2.744
intI1	integrase	MGE	5.762
intI1	integrase	MGE	2.396
intI1	integrase	MGE	0.706
IncP_oriT	plasmid incomp.	MGE	22.363
repA	plasmid replication	MGE	0.735
IS6 *tnpA*, IS26, ISG 6, Tn24, Tn25	transposase	MGE	0.395
IS6 Group, *tnpA*, IS6100, ISG 6	transposase	MGE	0.229
IS4 Group, *tnpA*, IS4, ISG 4, Tn22	transposase	MGE	448.695
IS21 Group, *tnpA*, Tn21, ISG 21	transposase	MGE	5.049
IS6 Group, IS1216	transposase	MGE	2.116
IS6 Group, *tnpA*	transposase	MGE	0.350
lmrA	MLSB	efflux	0.100
ermC	MLSB	protection	12.592
sul2	sulfonamide	protection	0.730
sul2	sulfonamide	protection	0.744
sul1	sulfonamide	protection	2.166
tetPB	tetracycline	protection	1.865
tetE	tetracycline	efflux	303.914

Table S4. Fold changes of ARGs calculated for R-sprinkler water, using D-sprinkler water as reference (sampling-1). Entries sorted by decreasing fold change. Equal abundance at fold change 0.053, indicated by horizontal line.

Gene Name	Target Antibiotics (Major)	Functional Classification	Fold Change
qacEΔ1	MDR	efflux	37.135
aadA	aminoglycoside	deactivate	32.050
aadA	aminoglycoside	deactivate	26.101
qacEΔ1	MDR	efflux	23.516
IS6 *tnpA*, IS26, ISG 6, Tn24, Tn25	transposase	MGE	23.343
aadA	aminoglycoside	deactivate	17.347
aadA	aminoglycoside	deactivate	9.885
qacEΔ1	MDR	efflux	8.978
strB	aminoglycoside	deactivate	7.226
qacH	MDR	efflux	6.889
sul1	sulfonamide	protection	6.144
IS4 Group, *tnpA*, IS4, ISG 4, Tn22	transposase	MGE	4.860
intI1	integrase	MGE	3.651
tolC	MDR	efflux	3.400
tolC	MDR	efflux	3.119
acrB	MDR	efflux	2.787
intI3	integrase	MGE	2.684
intI3	integrase	MGE	2.320
aadA	aminoglycoside	deactivate	1.910
blaCMY	β-lactam	deactivate	1.764
IS6 Group, IS1216	transposase	MGE	1.721
mdtF	MDR	efflux	1.545
aadA	aminoglycoside	deactivate	1.306
mdtH	MDR	efflux	1.287
IS6 Group, *tnpA*	transposase	MGE	1.153
tolC	MDR	efflux	1.125
blaCMY	β-lactam	deactivate	1.089
mdtL	amphenicol	efflux	1.072
blaTEM	β-lactam	deactivate	0.999
lmrA	MLSB	efflux	0.833
mdtE	MDR	efflux	0.800
aac3-VI	aminoglycoside	deactivate	0.735
acrA	MDR	efflux	0.678
acrR	MDR	regulator	0.573
bacA	other	deactivate	0.556
ampC	β-lactam	deactivate	0.543
KPC	β-lactam	deactivate	0.496
mdtG	MDR	efflux	0.440
tetE	tetracycline	efflux	0.337
qnrB	quinolone	protection	0.322
ampC	β-lactam	deactivate	0.313
acrA	MDR	efflux	0.301
ttgA	MDR	efflux	0.277
acrA	MDR	efflux	0.273

rarD	amphenicol	efflux	0.241
tetA	tetracycline	efflux	0.226
marR	MDR	regulator	0.222
bacA	other	deactivate	0.221
mepA	MDR	efflux	0.198
blaOXA	β-lactam	deactivate	0.171
intI1	integrase	MGE	0.159
intI1	integrase	MGE	0.155
aadA5	aminoglycoside	deactivate	0.143
yidY/mdtL	amphenicol	efflux	0.138
intI1	integrase	MGE	0.130
mexE	MDR	efflux	0.126
vanSC	vancomycin	protection	0.116
sul2	sulfonamide	protection	0.099
mexF	MDR	efflux	0.088
rarD	amphenicol	efflux	0.082
oprD	MDR	efflux	0.060
IncP_oriT	plasmid incomp.	MGE	0.036
blaFOX	β-lactam	deactivate	0.025
ISSm2	insertional seq.	MGE	0.022
repA	plasmid replication	MGE	0.015
merA	mercury	deactivate	0.008
ISPps	insertional seq.	MGE	0.007
IS6 Group, *tnpA*, IS6100, ISG 6	transposase	MGE	0.003
IS21 Group, *tnpA*, Tn21, ISG 21	transposase	MGE	0.0002
pAKD1	plasmid replication	MGE	0.0001
